# Biodegradation Pattern of Glycopolymer Based on D-Mannose Oligomer and Hydroxypropyl Acrylate

**DOI:** 10.3390/polym12030704

**Published:** 2020-03-22

**Authors:** Ana-Maria Pană, Valentin Ordodi, Gerlinde Rusu, Vasile Gherman, Geza Bandur, Lucian-Mircea Rusnac, Gabriela-Alina Dumitrel

**Affiliations:** 1 Faculty of Industrial Chemistry and Environmental Engineering, Politehnica University Timişoara, 6 Vasile Pȃrvan bvd., 300223 Timişoara, Romania; anamaria.pana@upt.ro (A.-M.P.); valentin.ordodi@upt.ro (V.O.); gerlinde.rusu@upt.ro (G.R.); geza.bandur@upt.ro (G.B.); lucian.rusnac@upt.ro (L.-M.R.); 2 Faculty of Civil Engineering, Politehnica University Timişoara, 2 Traian Lalescu Str, 300223 Timisoara, Romania; vasile.gherman@upt.ro

**Keywords:** glycopolymer, DSC, isoconversional methods, biodegradation, bioreactor, kinetic modeling, TG, FTIR

## Abstract

Glycopolymers are polymers with sugar moieties which display biodegradable and/or biocompatible character. They have emerged as an environmentally-friendly solution to classical synthetic polymers and have attracted significant research interest in the past years. Herein, we present the synthesis of a D-mannose based glycopolymer with biodegradable features. The glycopolymer was synthesized by radical copolymerization between a D-mannose oligomer bearing polymerizable double bonds and 2-hydroxypropyl acrylate, in a weight ratio of 1:2. The copolymerization kinetics was investigated by differential scanning calorimetry (DSC) and the activation energy of the process was comparatively assessed by Kissinger–Akahira–Sunose and Flynn–Wall–Ozawa methods. The obtained glycopolymer displayed good thermal behavior, fact proven by thermogravimetrical (TG) analysis and it was submitted to biodegradation inside a bioreactor fed with water from the Bega River as the source of microbial inoculum. The glycopolymer sample degraded by approximately 60% in just 23 days. The biodegradation pattern of the glycopolymer was successfully fitted against a modified sigmoidal exponential function. The kinetic model coefficients and its accuracy were calculated using Matlab and the correlation coefficient is more than promising. The changes inside glycopolymer structure after biodegradation were studied using TG and FTIR analyses, which revealed that the sugar moiety is firstly attacked by the microbial consortia as nutrient source for proliferation.

## 1. Introduction

Sugar derived polymers have emerged as an eco-friendly alternative to common plastic materials in the context of growing environment concern and escalating petroleum prices [1]. It is worth mentioning that plastics derived from petroleum are versatile materials [2] with good thermal and strength properties [3] with diverse applications: automotive industry, constructions, food packaging, health care, etc. [4]. Nonetheless, despite their low price and availability they have a major drawback, including economic loss of material value and environmental damage [5]. Over the years, impressive amounts of plastic debris have accumulated in the environment [6], mostly they are carried by water sources [7] and revealed to humanity as a giant flaw of industrial globalization [8].

Scientists from all around the world and governmental authorities are working to find a solution to this major pollution concern. The idea of recycling plastic materials is one of the very best [9], but it takes time to educate people as to reuse and rethink before they act. Also, plastic materials are being incinerated or deposited on landfills [10,11], however these treatment solutions are more environmentally invasive than recycling [12].

The main idea is to develop new competitive materials based on renewable feed-stocks [13,14] that could become feasible alternative to common plastics but with improved biodegradable features [15,16]. Biodegradation is the process of altering the structure of an organic based material under microorganisms’ attack [17]. The biodegradation process can be intensified if the material is found in the form of small sphere-like fragments, right humidity and temperature parameters and direct contact to the microbial source [18]. Other abiotic factors can influence the biodegradable features of a material: light, wind, moist, mechanical scission, etc. [19].

Sugar based polymers have the potential of degradation under biotic factors as microorganism may have the skills to recognize the sugar skeleton and to use it as carbon source [20,21]. Thus, glycopolymers can emerge as plastic materials with the same good strength and thermal stability but with less impact onto environment, with applications in medicine, agriculture, food packaging, etc. [22,23,24,25,26].

Our research group has been involved in the modification of the sugar skeleton for obtaining competitive polymeric materials with biodegradable features. We have focused both on monosaccharides [27,28,29,30,31,32] and on polysaccharides [33,34,35] and managed to obtain and characterize new glycopolymers for common applications and biomedicine. Recently, we have been involved in the development of new strategies for eco-friendly degradation of plastic materials [36,37]. In this regard, our team has designed a bioreactor for the biodegradation of sugar based polymers in a controlled environment, using pure microorganism cultures or natural bacterial consortia, in aerobic or anaerobic atmosphere. The working parameters were monitored continuously and the kinetics of the process was assessed by weighing the polymeric samples from time to time.

The work herein presents the biodegradation pattern of a glycopolymer derived from a D-mannose oligomer with maleic backbone and 2-hydroxypropyl acrylate inside the bioreactor. The glycopolymer was obtained by radical copolymerization process, which was kinetically investigated herein using two isoconversional methods. The thermal analysis of the glycopolymer samples before and after biodegradation revealed that the sugar skeleton was mainly attacked by the microorganisms, but also the cleavage of acrylic chain was visible as the sample deteriorated after incubation. The aerobic biodegradation process was modeled using a kinetic mathematical expression that accurately describes the process.

## 2. Materials and Methods 

D-mannose based oligomer (M) was isolated in our laboratory previously [17]. 2-hydroxypropyl acrylate (HPA) and benzoyl peroxide (BOP) were purchased from Merck (Merck KGaA, Darmstadt, Germany) and used without purification. All other reagents were used as purchased. 

### 2.1. Glycopolymer Synthesis

The D-mannose oligomer was obtained according to a protocol described elsewhere [28,38]. The copolymerization process consisted in homogenous dissolution of the M in HPA (weight ratio 1:2, herein referred to as M_HPA2), then the addition of the radical initiator was done – benzoyl peroxide (BOP) 1 wt.% from the mixture. The mixture was stirred for about a half an hour until perfect homogeneity. Samples of about 4 mg were collected for differential scanning calorimetry (DSC) analysis and then this mixture was poured into glass vials and heated gradually by 10 °C/h until 120 °C. The obtained glycopolymer samples were taken out of the vial, analyzed by thermogravimetrical (TG), FTIR, and tested for biodegradation.

### 2.2. DSC Analysis

The copolymerization process kinetics of D-mannose derived oligomer with 2-hydroxypropyl acrylate (HPA) in weight ratio of 1:2 was studied by DSC. The DSC diagrams were registered on a Netzsch 204 device (NETZSCH-Gerätebau GmbH, Selb, Germany), in nitrogen atmosphere, on a heating range of 20–200 °C and considering 5 different heating rates: 2.5, 5, 7.5, 10, and 20 °C/min. The homogeneous mixture of M: HPA (weight ratio 1:2) containing benzoyl peroxide (1% weight) as radical polymerization initiator, was placed in liquid form (4.0 ± 0.1 mg) in closed aluminum crucibles and analyzed according to the established temperature program.

### 2.3. Kinetic Isoconversional Methods for Activation Energy Assessment 

Kissinger–Akahira–Sunose (KAS) is a model-free (isoconversional) method for kinetic assessments. The mathematical expression involves the linear dependence between ln($\beta_{i}/T_{\alpha i}^{2})\;$ and 1/T_αi_, based on the equation:

(1) $$ln\frac{\beta_{i}}{T_{\alpha i}^{2}}=-\frac{E_{\alpha }}{RT_{\alpha i}}+const.$$

where: the subscript i denotes different heating rates, β is the heating rate (K/min), α is the conversion degree (conversions ranging between 10% and 90%), E_α_ is the activation energy at the given conversion (kJ/mol), T_αi_ is the absolute temperature at considered conversion and heating rate (K), R is the gas constant, R = 8.314 J/K mol and const. is a constant value.

The Flynn-Wall-Ozawa (FWO) isoconversional method of assessing the activation energy of a process at different heating rates and conversion represents one of the first integral mathematical models. The mathematical relation which allows the calculation of activation energy from the slope of the linear plot ln(β_i_) against 1/T_αi_ is:

(2) $$ln\beta_{i}=const.-1.052\left(\frac{E_{\alpha }}{RT_{\alpha i}}\right)$$

where: the subscript i denotes different heating rates, β is the heating rate (K/min), α is the conversion degree (conversions ranging between 10 and 90%), E_α_ is the activation energy at the given conversion (kJ/mol), T_αi_ is the absolute temperature at considered conversion and heating rate (K), R is the gas constant, R = 8.314 J/K mol [39] and const. is a constant value.

### 2.4. Thermogravimetry

In order to establish the thermal stability, the glycopolymer was submitted to a thermogravimetrical (TG) analysis. 4.0 ± 0.15 mg of samples, before and after biodegradation were weighted in open alumina crucibles (average mass 190 ± 1.0 mg) in a TG 209 F1 Libra (NETZSCH-Gerätebau GmbH, Selb, Germany) device having high resolution (0.1 μg), in nitrogen atmosphere. Curves were recorded on a temperature range of 20–500 °C and a heating rate of 10 °C/min.

### 2.5. ATR-FTIR

FTIR spectra of the glycopolymer samples before and after biodegradation process were recorded using Bruker Vertex 70 spectrometer with ATR (Bruker Optics Gmbh, Ettlingen, Germany), at room temperature. The samples were analyzed in the range 3800–500 cm^−1^.

### 2.6. Biodegradability Testing

An aerobic bioreactor was used to test the glycopolymer biodegradability in natural aqueous media from the Bega River which crosses Timişoara. The active microorganisms detected in the Bega River were three groups of ecophysiological microorganisms: nitrifying bacteria, fixing bacteria (aerobic – *Azotobacter vinelandii* and *Azotobacter chroococcum*, respectively anaerobic – *Clostridium sp*.) and iron-reducing bacteria [40], but also microorganisms, viruses and protozoa derived from anthropogenic sources (coliforms) [41,42]. The glycopolymer samples in the form of discs (46 mm diameter) were placed in the laboratory scale bioreactor fed with water from the Bega River. The bioreactor was designed and operated by our research team; it was provided with thermoset unit, stirring device, aeration pump and ports for sampling. The incubation process occurred at 37 ± 0.5 °C for 23 days, maintaining an air flow of 2 L/min. The polymer samples were weighted from time to time in order to assess the weight loss (3) during the biodegradation process.

(3) $$\%Weight\;loss=\left(\frac{w_{0}-w}{w_{0}}\right)\times 100$$

The microbiological environment was characterized by colony forming units of Gram positive and negative bacteriological assay, at the beginning and at the end of the process. After biodegradation process, the samples were taken out of the vial, washed with water and ethanol for several times for removal of bacteria/fungus residues, then air dried until constant weight. The experiments were run in duplicate.

### 2.7. Mathematical Models for Kinetics Modeling of Biodegradation Process

The mathematical model used to better understand the aerobic biodegradation of polymers in aqueous media was a modified sigmoidal exponential function (4) [43]:

(4) $$W\left(t\right)=\left(\frac{100\cdot a_{1}}{a_{2}+a_{4}+a_{6}}\right)\cdot \left(\frac{a_{1}+a_{2}+a_{4}+a_{6}}{a_{1}+a_{2}\cdot e^{-a_{3}\cdot t}+a_{4}\cdot e^{-a_{5}\cdot t^{2}}+a_{6}\cdot e^{-a_{7}\cdot t^{3}}}-1\right)$$

where: W – weight loss (%) at time t, a_1_, a_2_, a_3_, a_4_, a_5_, a_6_, a_7_ – constants. 

This expression take into account the following assumptions: the cumulative weight loss during time cannot be negative; the biodegradation process, expressed as weight loss, is nonlinear with time; the weight loss at zero time is zero and by infinite time is maximum. 

For calculation of model parameters, a non-linear unconstrained optimization method was used together with the Nelder–Mead algorithm, which minimizes a scalar-valued nonlinear function of n real variables, by using only function values [44].

The software used was MATLAB R2018a Software Package (The MathWorks, Inc., Massachusetts, United States). The model accuracy was estimated graphically and by calculating the following evaluation parameters: the relative absolute error (rAE), correlation coefficient (R), determination coefficient (R^2^), mean square error (SD), and the root mean square error (RMSE). 

## 3. Results

The idea of renewable raw materials for polymers came along their lack in degradability after disposal. The plastic materials industry is based on large market of diverse objects, most of them of single use, which cannot be easily degraded and which persisted in the environment for large periods. The glycopolymers we have synthesized prior have proved as good alternatives for common plastics, due to their good thermal behavior and plasticity but they have displayed biodegradability when tested *in vitro*, using pure cultures [30,36], or natural occurring bacteria consortia [37]. 

### 3.1. Glycopolymer Synthesis. DSC Kinetic Analysis

**Scheme 1:**
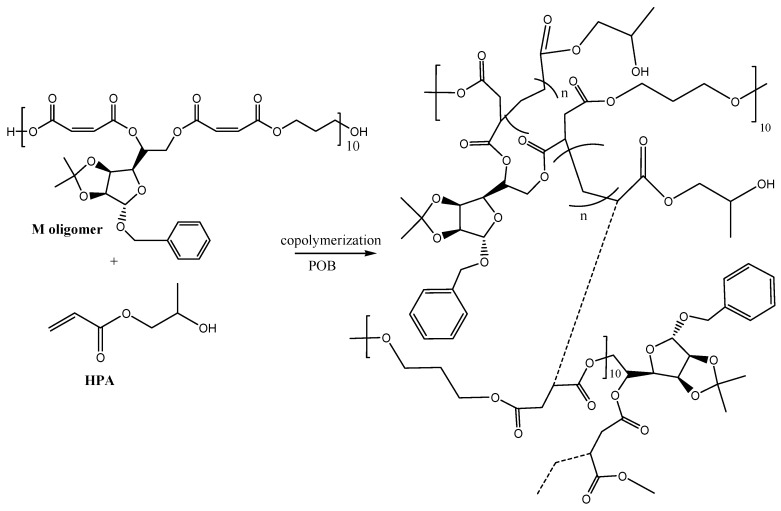
The copolymerization reaction scheme between the D-mannose oligomer (M) and 2-hydroxypropyl acrylate (HPA).

This present work presents the synthesis of a glycopolymer based on natural raw material, i.e., the D-mannose oligomer (M) and a common monomer, 2-hydroxypropyl acrylate (HPA). The D-mannose oligomeric structure was previously investigated by NMR and FTIR spectroscopy, while the HPLC-ESI-MS analysis revealed the presence of 10 repeating units inside the oligomeric sugar chain. The M oligomer presents double bonds susceptible for copolymerization provided by the maleic moieties and solubility in HPA in weight ratio of 1 to 2. The copolymerization process was carried out in bulk, following the complete dissolution of the M oligomer into the acrylic co-monomer. The copolymerization process is illustrated in Scheme 1. The C=C double bonds from the maleic moiety and those from the acrylic monomer form new C–C bonds along the sugar oligomer chain during the radical copolymerization initiated by the BOP promoter, turning into a crosslinked polymeric network. 

**Figure 1:**
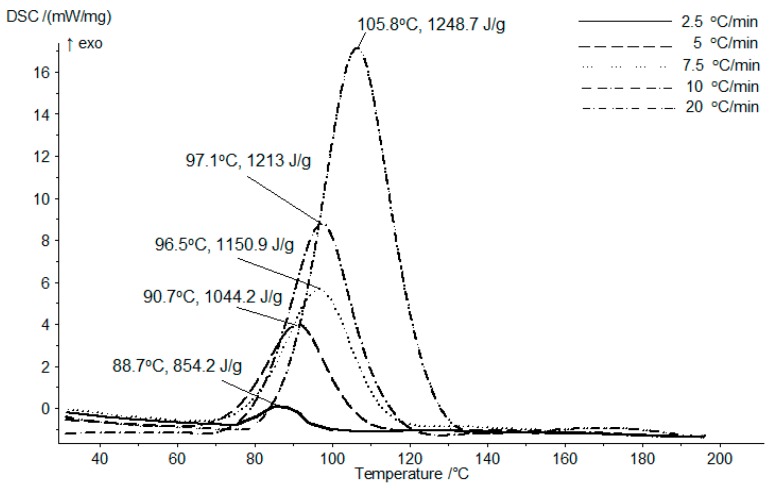
The differential scanning calorimetry (DSC) diagram for the copolymerization process between the D-mannose oligomer and HPA, weight ratio 1:2.

The copolymerization process of M oligomer and HPA in weight ratio of 1 to 2 was kinetically investigated following a differential scanning calorimetry (DSC) technique using several heating rates. Figure 1 presents the DSC diagram for the heating rates considered, on the heating range of 20 to 200 °C. The copolymerization process occurs in one-step, fact emphasized by the presence of a single peak. The peak temperature increases along the heating rate increase, displaying values on the temperature range between 88 and 107 °C. Also, the exothermic energy necessary to complete the copolymerization process, increased with the growth in heating rate; the peak area energy registered values in the range 850 and 1250 J/g. 

**Figure 2:**
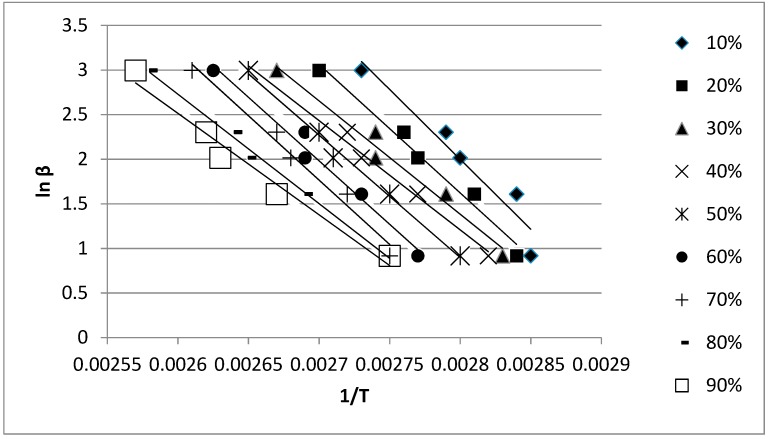
Flynn–Wall–Ozawa linear dependencies for the copolymerization process between D-mannose oligomer and 2-hydroxypropyl acrylate (weight ratio 1:2).

The activation energy of the copolymerization process between the mannose oligomer and HPA in weight ratio of 1 to 2 was assessed by applying two isoconversional methods: Kissinger–Akahira–Sunose and Flynn–Wall–Ozawa. The methods imply the linear dependence between the natural logarithm of the heating rate divided by the square of the temperature and the natural logarithm of the heating rate, respectively versus the temperature’s reverse. Figure 2 presents the Flynn–Wall–Ozawa linear dependencies obtained for conversions ranging from 10% to 90% during the copolymerization process. The temperatures corresponding to the considered conversions were obtained by slicing the peak area into areas proportional to the respective conversion. The lines were obtained by running the copolymerization process inside the DSC using 2.5 °C/min, 5 °C/min, 7.5 °C/min, 10 °C/min, and 20 °C/min heating rates. Similar linear dependencies were registered by using the Kissinger–Akahira–Sunose method. The correlation coeficient for all the resulting linear dependences displayed values above 0.95.

Table 1 comparatively presents the values of the activation energy for the copolymerization process obtained using both isoconversional methods described above. The activation energy of the copolymerization process generally increases as the conversion increases, and the average activation energy of the process can be assessed as the arithmetic average of all calculated values. The values of the average activation energy for the copolymerization process is similar for both isoconversional methods applied herein. 

### 3.2. Biodegradation Studies: Kinetics

Due to its good polymeric features, the material was tested for biodegradability using a bioreactor equipped with air pump and continuous stirring designed in our laboratory. Our group has performed in the past *in vitro* biodegradation experiments, in test tubes, using pure microbial cultures (*Zymomonas mobilis* and *Trichoderma reesei*) [30] and natural occurring consortia [37] in order to acknowledge the susceptibility of sugar derived polymers for degradation under biotic factors.

The experiment presented herein represents a step forward as it envisions the possibility of industrial decomposition of polymers under microorganisms’ action using bioreactors. The bioreactor fed with water from the Bega River was designed to develop the microorganism culture under aerobic factors, following a thermoset temperature of about 37 °C, prolific to the development of microbial consortia. It is noteworthy that no other nutrient source was added to the environment as the microorganisms are forced to consume the glycopolymer as carbon source. 

The weight loss profile of the glycopolymer sample followed for about 23 days reveals that the material alteration is basically dependent on the development of the microbial environment, following a lag phase of about 1–2 days corresponding to the growth of the bacterial consortia, then the proliferation phase, leading to the total weight loss of about 60%. The colony forming units of the water from the Bega River was assessed and it revealed that the total amount of Gram positive microorganisms reached about 18,000 units, while the Gram negative surpassed 85,000, which sumed up to over 100,000 units per mL. Before the biodegradation process the microbiological assay revealed a total of about 30,000 colony forming units, thus the bioreactor was a useful equipment for the good proliferation of bacteria from the Bega River.

**Figure 3:**
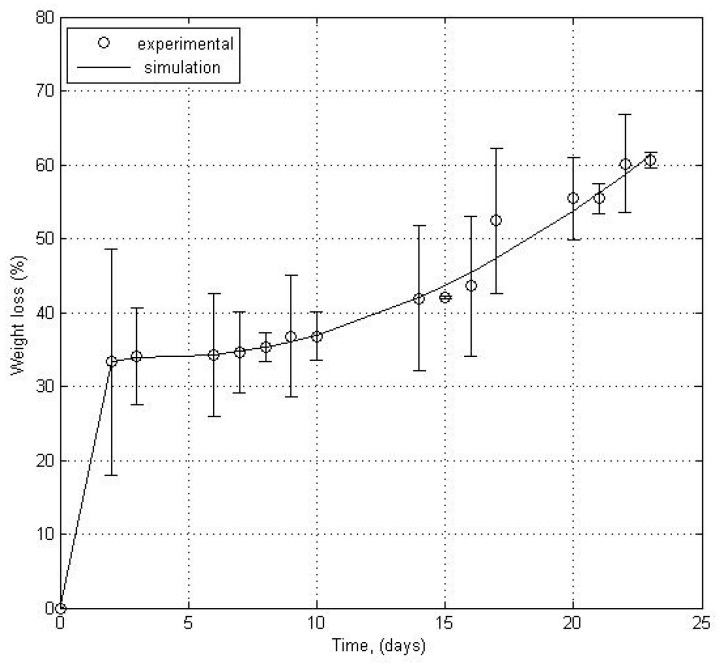
Experimental and predicted polymer weight loss profiles during biodegradation in water from the Bega River.

The biodegradation process was described from kinetics point of view using a mathematical model, namely a modified sigmoidal exponential function. Figure 3 presents the experimental weight loss data calculated as the mean of the two experimental replicates against the predicted ones modeled according to Equation (4). The bars that indicate the standard error of the mean were also represented. The values of kinetic model parameters, calculated by fitting it with the experimental data were presented in Table 2.

The modified sigmoidal exponential function, used generally to describe the proliferation of bacteria populations, was appropriate to describe the biodegradation process of the glycopolymer based on D-mannose oligomer and 2-hydroxypropyl acrylate. This fact emphasized the dependency between the biodegradation susceptibility of the glycopolymers and the growth of the bacterial consortia. The values of parameters used to estimate the model accuracy were presented in Table 3.

The values from Table 3 revealed the fact that the model described very well the phenomena that occurred during polymer biodegradation in aqueous media. Based on the mathematical model, it was possible to predict the biodegradation behavior of the polymer in the aqueous media: a half-life of 18.32 days, a lifetime of 64.1 days, and a lag phase of 1 day. Considering the accuracy of this model, the expected lifetime for the glycopolymer inside the bioreactor is promising, considering that most plastic material end up in water. That would mean that if this glycopolymer would be used as packaging material and would be disposed after use unintentionally in the Bega River, the conditions for its total breakdown in less than six months are most likely to happen. By comparison, other biodegradable materials are more likely to be decomposed in about 16–24 weeks in natural environment consisted of fresh water sources [45]. Nonetheless, without proper aeration and stirring provided inside the bioreactor, the degradation kinetics of the glycopolymer would be slower and more susceptible to temperature fluctuations.

By losing 60% of its weight in 23 days of incubation inside a bioreactor fed with natural inoculum from the Bega River, the glycopolymer displayed good biodegradation susceptibility. Also, the degradation pattern of the sample was dependent on the proliferation phase of the microbial consortia, the lag phase in the development of the bacteria corresponding to the adjustment to their bioreactor conditions being easily identified in its weight loss profile.

From structural point of view, the behavior of the glycopolymer sample before and after the incubation into microbial rich environment was investigated using FTIR and TG analyses.

### 3.3. TG Analysis of Glycopolymer Sample before and after Biodegradation 

**Figure 4:**
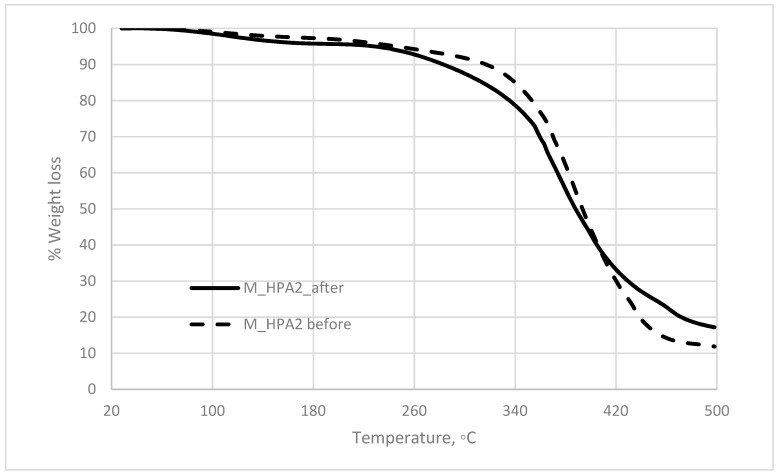
The thermogravimetrical (TG) diagram for the glycopolymer before and after biodegradation.

The polymeric material isolated after the copolymerization process was analyzed using the thermal techniques. M_HPA2 glycopolymer displayed good resilience to thermal treatment as the TG diagram (Figure 4) confirmed. When analyzing the thermal stability on certain temperature ranges, it could be noticed that the material was very stable at temperatures below 200 °C, losing less than 1% when heated to 100 °C and about 3% up to 200 °C. For the thermal behavior between 20 and 300 °C, the weight loss was about 8.5%, while up to 400 °C more than half of the total weight sample was degraded. The total weight loss on the analyzed temperature range was about 88% (Table 4). 

The thermal stability of the glycopolymer sample after biodegradation (Figure 4) revealed that overall the sample was less susceptible to higher temperatures, by losing less than 83% up to 500 °C, but nonetheless on the temperature ranges up to 100, 200, or 300 °C the weight loss was significantly higher than the non-biodegraded sample. On the heating range of 20 to 200 °C, the glycopolymer sample before biodegradation had lost about 3% of its weight, while after biodegradation the loss was about 4.3% (Table 4). The same tendency was registered on the range of 30 to 300 °C, with 8.25% for the original sample and more than 12.5% weight loss for the biodegraded one. This difference could be explained by the fact that microorganisms by their metabolism may have metabolized some of the sugar skeleton, and at the same time have altered the larger polymeric molecules and broken them into smaller moieties more susceptible to thermal treatment. 

Moreover, the TG curves had different allures, as their inflexion points (not shown in Figure 4) are shifted to lower temperatures up to 400 °C, than to higher temperatures (Table 5). Also, a new inflexion point was detected at about 358 °C, which the sample of glycopolymer before biodegradation did not present. This tendency was probably caused by the alteration of the cross-linked polymeric chain, which was hindered by the presence of the microorganisms entering the polymeric network. It is expected that the microorganisms to consume the available sugar moieties from the mannose oligomer at first and then to turn to the acrylic chains. The consortia were expected to present enzymes able to lyse the esteric bond found both in the sugar oligomer and the acrylate and to extract smaller molecules that would be used as carbon source. That is why, after the biodegradation, when only about 40% of the original weight was left from the original polymeric network the chains were more susceptible to thermal treatment at lower temperatures but overall, because most acrylic part was still in the sample, the residual weight was higher than beforehand.

### 3.4. FTIR Analysis of Glycopolymer Sample before and after Biodegradation 

**Figure 5:**
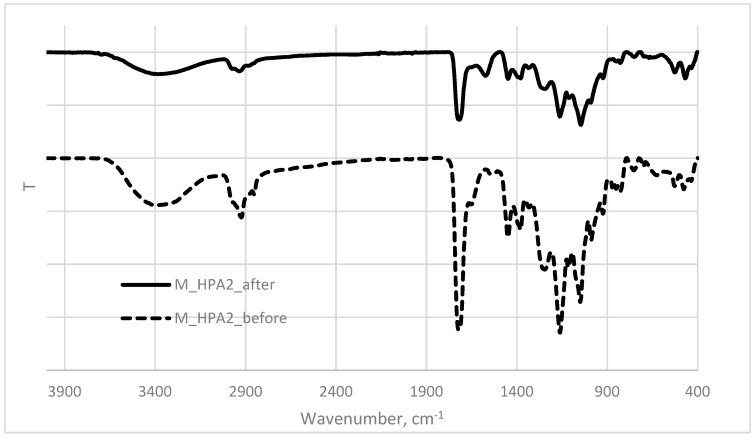
The superimposed FTIR spectra of the glycopolymer sample before and after biodegradation.

The alteration of the polymeric network after biodegradation was emphasized also by the FTIR analysis (Figure 5). The FTIR spectrum for the glycopolymer sample after biodegradation was neater and the vibration bands were less intense [37]. The associated OH vibration band from about 3430 cm^−1^ was shifted to higher wavenumber at about 3448 cm^−1^, indicating that the molecules from within the polymer matrix were drifted at larger distances because of microorganisms attack. The vibration bands corresponding to the methylene and methyl groups from the sugar skeleton with intense peak at about 2944 cm^−1^ were less intense than before, while the aromatic groups from about 3010 cm^−1^ displayed a more intense vibration pattern in the degraded sample. The strong vibration band corresponding to the C=O esteric group from about 1730 cm^−1^ was clearly identified in the spectra run before and after biodegradation, certifying that the sugar oligomer was firstly consumed and then the acrylic polymeric chain [30,36].

## 4. Conclusions

Glycopolymers synthesized herein based on D-mannose oligomer and 2-hydroxypropyl acrylate in a weight ratio of 1:2 were obtained by radical copolymerization. The kinetics of the copolymerization process were studied and the activation energy was assessed using two isoconversional methods: Kissinger–Akahira–Sunose and Flynn–Wall–Ozawa. The obtained glycopolymer sample was tested for biodegradation inside a self-designed aerobic bioreactor, at thermoset temperature under continuous stirring for 23 days. The biodegradation process was investigated following a weight loss profile, which indicated good fitting against a modified sigmoidal exponential function. According to the model, the glycopolymer will be completely decomposed in about 2 months. The changes of the crosslinked polymeric network were identified by TG and FTIR analyses, which indicated that the sugar skeleton from the sample is recognized at first by the bacteria and consumed as carbon nutrient source and then the lyse of the acrylic chains occur. By degrading with about 60% of their weight in just 23 days inside the bioreactor fed with available, cost-free bacteria from the Bega River, the experiment could be of use for implementation on a larger scale and on different glypolymeric substrates.

## Figures and Tables

**Table 1:** The activation energies calculated using the isoconversional methods Kissinger–Akahira–Sunose and Flynn–Wall–Ozawa.

Conversion, %	Activation Energy Ea, kJ/mol	
	Kissinger-Akahira-Sunose	Flynn–Wall–Ozawa
10	88.78	89.84
20	94.85	96.94
30	95.02	96.82
40	98.74	100.42
50	106.40	108.45
60	110.95	112.41
70	112.10	113.97
80	113.54	114.2
90	118.95	120.78
Average Activation Energy, kJ/mol	104.37	105.98

**Table 2:** Model parameter values.

Parameter	a_1_	a_2_	a_3_	a_4_	a_5_	a_6_	a_7_
	0.04478	−0.0222	0.1296	0.1048	0.0024	1764.2996	1.6623

**Table 3:** Parameters values used to estimate the model accuracy.

Root-Mean Square Error, RMSE	Mean Square Error SD	Determination Coefficient R^2^	Correlation Coefficient R	Relative Absolute Error, rAE
1.5534	2.4133	0.9987	0.9942	0.0015

**Table 4:** Weight loss of the glycopolymer before and after biodegradation.

Sample	Weight Loss, %				
	20–100 °C	20–200 °C	20–300 °C	20–400 °C	20–500 °C
M_HPA2_before	0.9573	3.0940	8.2544	56.0916	88.1286
M_HPA2_after	1.4948	4.3422	12.5916	57.4605	82.7941

**Table 5:** Inflexion points of TG curves before and after biodegradation.

Sample	Inflexion Point Temperature, °C			
M_HPA2_before	-	368.8	386.5	434.3
M_HPA2_after	358	363.8	377.8	462.9
